# Impact of Indocyanine Green (ICG) Fluorescence Cholangiography on Operative Time and Safety in Laparoscopic Cholecystectomy: A Prospective Randomized Controlled Study

**DOI:** 10.7759/cureus.88639

**Published:** 2025-07-24

**Authors:** Sithdharthan Ravikumar, Raju Musham, Ganta V Vardhan Rao, Vimalakar R Eppa

**Affiliations:** 1 General Surgery, KIMS-Sunshine Hospitals, Hyderabad, IND; 2 General Surgery, George Eliot Hospital, Nuneaton, GBR; 3 Surgical Gastroenterology, KIMS-Sunshine Hospitals, Hyderabad, IND

**Keywords:** • cbd: common bile duct, • cvs: critical view of safety, • gb: gallbladder, • icg: indocyanine green, • lc: laparoscopic cholecystectomy, • oc: open cholecystectomy

## Abstract

Background

Laparoscopic cholecystectomy (LC) is the gold standard for treating gallbladder disease; however, bile duct injury (BDI) remains a significant concern. Indocyanine green (ICG) fluorescence cholangiography has emerged as a real-time, radiation-free technique to enhance biliary visualization and reduce surgical risks. This study evaluates the impact of ICG fluorescence imaging on LC outcomes compared to conventional methods.

Aims and objectives

This study aims to evaluate the effectiveness of ICG fluorescence cholangiography in improving intraoperative biliary visualization during LC. The objectives include comparing operative time between ICG-assisted and standard LC, assessing the incidence of bile duct injuries and conversions to open surgery, evaluating intraoperative complications such as bleeding and bile leaks, and analyzing postoperative recovery parameters, including hospital stay duration.

Methods

A prospective randomized controlled study was conducted at Sunshine Hospital, Secunderabad, India, from December 2021 to April 2022. One hundred patients undergoing LC were randomized into two groups: ICG-assisted LC (n = 50) and standard LC (n = 50). ICG (0.5 mL) was administered intravenously one hour preoperatively, and fluorescence imaging was performed intraoperatively. Primary outcomes included operative time, BDI rates, conversion to open surgery, and overall surgical safety. Data were analyzed using SPSS, with a p-value < 0.05 considered statistically significant.

Results

ICG fluorescence cholangiography significantly reduced operative time (mean: 42.6 ± 5.2 min vs. 48.3 ± 6.1 min; p = 0.002). No bile duct injuries or conversions to open surgery were reported. Excessive bleeding (>100 mL) occurred in six cases (four in the ICG group, two in the non-ICG group; p = 0.48). Hospital stay was shorter in the ICG group (median: 1 day, range: 1-2 days) than in the non-ICG group (median: 1.3 days, range: 1-2 days; p = 0.045). No postoperative bile leaks or ICG-related complications were observed.

Conclusion

ICG fluorescence cholangiography significantly improves intraoperative biliary visualization, reduces operative time, and enhances surgical efficiency without increasing complications. These findings support its routine use in LC, although further research is needed to standardize protocols and assess its role in complex cases.

## Introduction

Laparoscopic cholecystectomy (LC) is one of the most commonly performed surgical procedures, with over a million cases conducted annually worldwide [1]. It is considered the gold standard for treating symptomatic gallbladder disease due to its minimally invasive nature and reduced postoperative recovery time [2]. However, despite its advantages, LC carries an inherent risk of complications, including bile duct injury, hepatic artery injury, and biliary leaks, with an incidence of 0.5-3% in LC compared to 0.1-0.5% in open cholecystectomy [3]. While minor bile duct injuries may be manageable, severe injuries can lead to long-term morbidity and necessitate complex reconstructive procedures [4,5].

To mitigate these risks, various techniques have been developed to enhance intraoperative visualization of the biliary anatomy. The Critical View of Safety (CVS), introduced by Strasberg SM et al. [6], is widely employed as a standard approach to ensure safe ductal identification before division. Additionally, intraoperative imaging techniques such as laparoscopic ultrasound and intraoperative cholangiography (IOC) have been utilized to improve surgical accuracy [7,8]. Despite these measures, bile duct injuries continue to pose a significant challenge in LC.

Indocyanine green (ICG) fluorescence imaging has emerged as a promising tool for intraoperative biliary visualization [9]. ICG is a water-soluble tricarbocyanine dye that binds to plasma proteins after IV administration and is exclusively excreted into the bile [10,11]. Its fluorescence, detectable in the near-infrared spectrum (790-805 nm), allows for enhanced real-time visualization of the biliary tree when captured using specialized infrared cameras [9,12]. This technique provides a non-invasive and dynamic assessment of biliary anatomy, potentially reducing the risk of iatrogenic injury.

Recent studies have demonstrated that ICG fluorescence cholangiography facilitates earlier and clearer identification of bile ducts, offering an advantage over conventional imaging modalities [13,14]. Moreover, ICG-assisted visualization has shown promise in complex cases, particularly in patients with severe inflammation or anatomical variations [15].

This study aims to evaluate the role of ICG fluorescence cholangiography in LC and compare its outcomes with non-ICG procedures. By assessing its impact on bile duct injury rates, operative time, conversion to open surgery, and overall surgical safety, this study seeks to determine whether ICG should be incorporated as a routine adjunct in LC.

## Materials and methods

Study design and setting

This randomized controlled study was conducted in the Department of Surgical Gastroenterology at Sunshine Hospital, Secunderabad, India, to assess the effectiveness of ICG fluorescence cholangiography in LC. The study spanned from December 2021 to April 2022, with a one-month follow-up period for all participants. A prospective, randomized, double-blinded design was employed to ensure an unbiased evaluation of ICG fluorescence cholangiography in LC. Patients were randomly assigned to either the ICG fluorescence group or the standard LC group using a computer-generated simple randomization technique.

To minimize bias, both the operating surgeon and outcome assessors were blinded to patient group assignments. Statistical comparisons were performed using independent t-tests for continuous variables (e.g., operative time, hospital stay duration) and chi-square tests for categorical variables (e.g., BDI rates, conversion to open surgery). All p-values were two-tailed, and a value of p < 0.05 was considered statistically significant. Comparisons were made between ICG-assisted and standard LC groups.

Study population

The study included all patients undergoing LC within the specified timeframe, with eligibility determined based on predefined inclusion and exclusion criteria. Patients diagnosed with benign gallbladder disease requiring elective or emergency LC were included, provided they gave informed written consent.

Exclusion criteria comprised patients unfit for surgery due to severe comorbidities or contraindications to laparoscopic procedures. Additionally, individuals with a known allergy to ICG were excluded, as were breastfeeding mothers due to the potential unknown effects of ICG excretion in breast milk.

Ethical considerations

Ethical approval was obtained from the Institutional Review Board (IRB) before patient recruitment commenced. The study adhered to international ethical guidelines for human research. Participants received comprehensive information regarding the study’s purpose, methodology, potential risks, and benefits. Written informed consent was obtained from all patients to ensure voluntary participation and adherence to ethical standards.

Study design and surgical technique

A total of 100 patients scheduled for LC were randomly divided into two groups: the ICG group (n = 50), which underwent LC with ICG fluorescence cholangiography for real-time intraoperative visualization of biliary anatomy; and the non-ICG group (n = 50), which underwent standard LC without ICG imaging.

To ensure consistency and minimize variability, all procedures were performed by a single experienced laparoscopic hepatobiliary surgeon. This standardized approach facilitated an accurate comparison between groups, with primary outcomes including operative time, BDI rates, conversion to open surgery, and overall surgical safety.

Data collection and statistical analysis

Patient data were systematically recorded in Microsoft Excel, encompassing demographic details, intraoperative findings, postoperative recovery parameters, and complications. The data were analyzed using SPSS software to compare outcomes between the ICG and non-ICG groups. The primary focus was to assess the impact of ICG fluorescence cholangiography on operative time, surgical safety, efficiency, and postoperative recovery.

This structured methodology ensured a rigorous and standardized evaluation of ICG fluorescence cholangiography in LC, providing reliable findings that could inform future clinical guidelines and best practices in laparoscopic surgery.

Operative techniques

**Figure 1:**
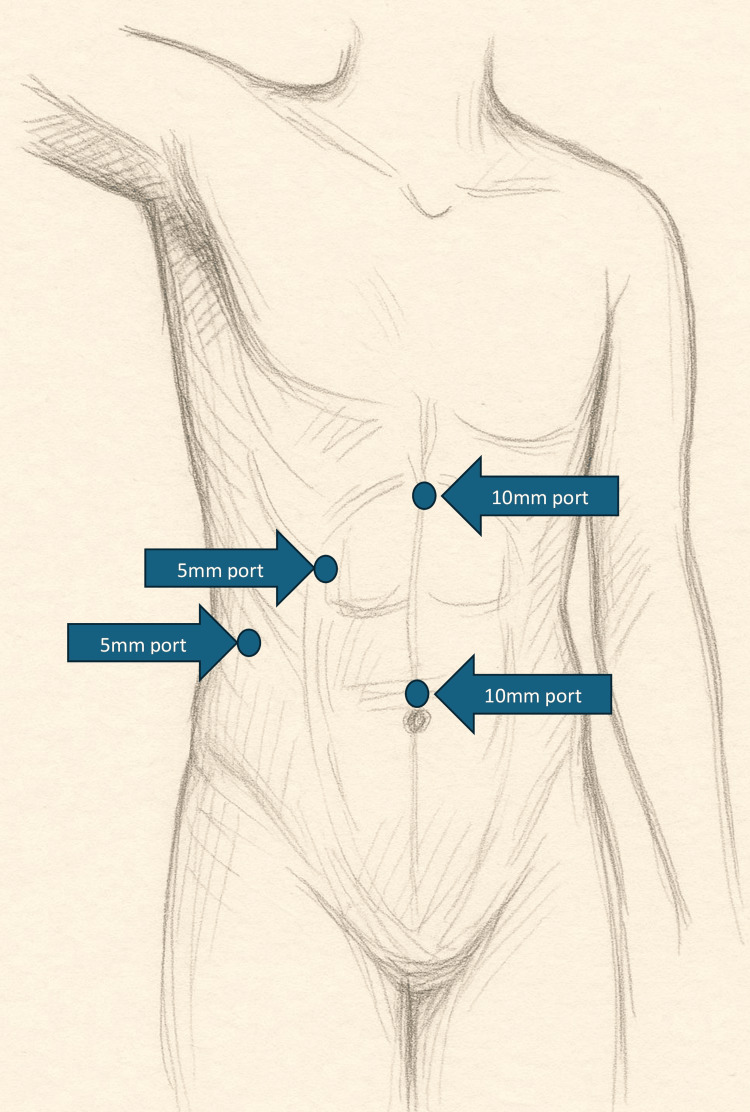
Patient and port positioning.

The standard four-port technique was utilized for LC, consisting of a 10 mm camera port and three 5 mm working ports. In cases where ICG fluorescence cholangiography was employed, a 0.5 mL dose of ICG was administered via intravenous infusion one hour prior to surgery (Figure 1).

Intraoperative fluorescence imaging was conducted using an endoscopic fluorescence imaging system, allowing enhanced visualization of the biliary tree. The procedure began with the exposure of Calot’s triangle, followed by meticulous dissection to identify and isolate the cystic duct and cystic artery. Although ICG was administered one hour preoperatively, fluorescence became visible intraoperatively within a few minutes of dissection, enabling early identification of the cystic duct and facilitating safer dissection. Once clearly visualized, both the cystic duct and artery were securely clipped and divided.

**Figure 2:**
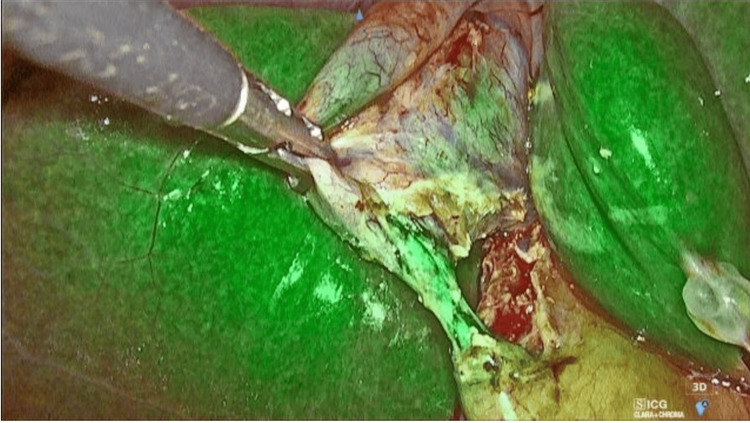
ICG image showing demarcation of the biliary tree. ICG: Indocyanine green.

**Figure 3:**
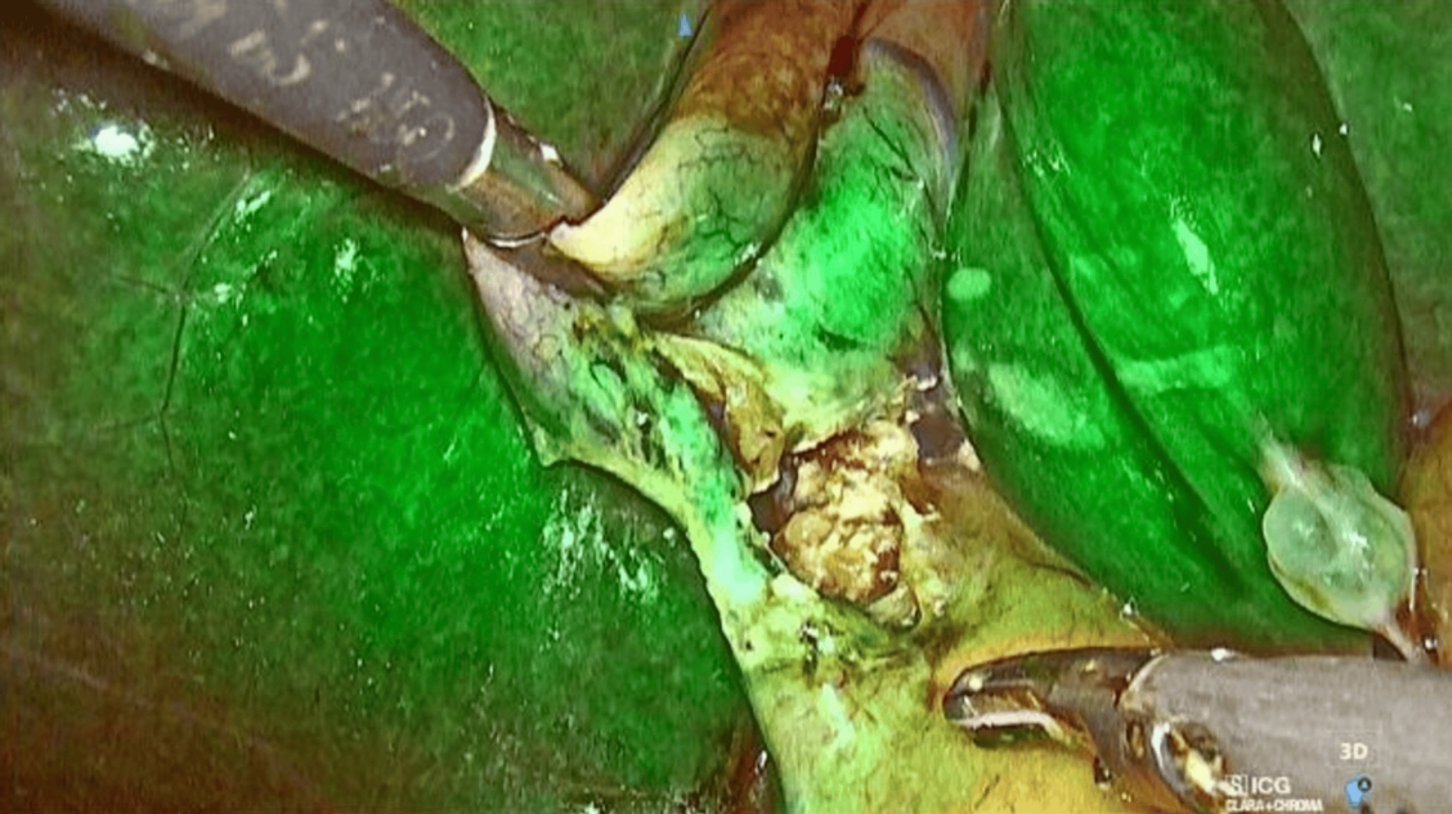
Dissected Calot's triangle visualized with ICG fluorescence. ICG: Indocyanine green.

**Figure 4:**
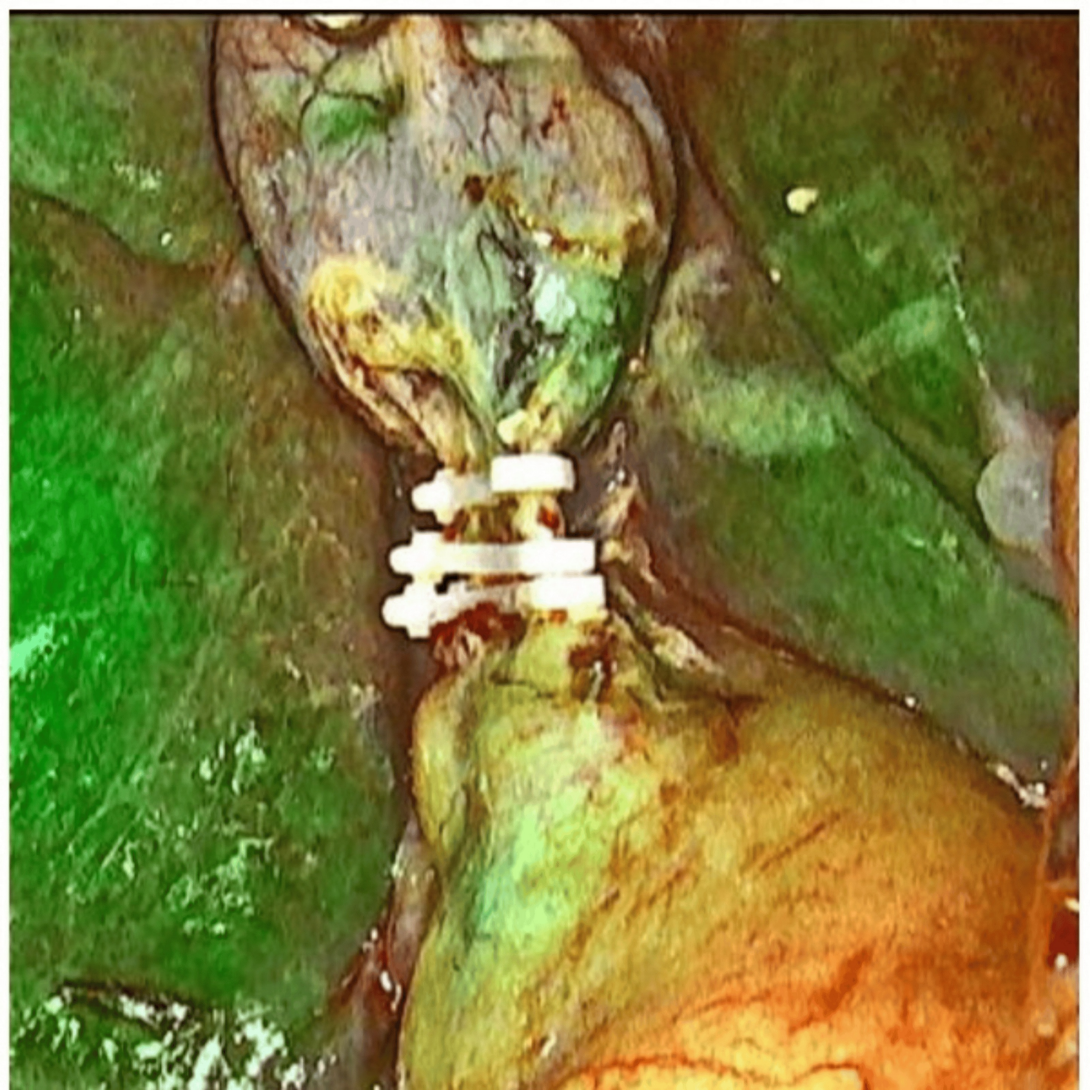
Cystic duct clipped under ICG guidance. ICG: Indocyanine green.

Subsequently, the gallbladder was carefully dissected off the liver bed following the standard technique. The specimen was then retrieved through the 10 mm port. While surgical drains were not routinely used, they were selectively placed in cases involving difficult dissections or increased intraoperative risk (Figures 2-4).

## Results

**Figure 5:**
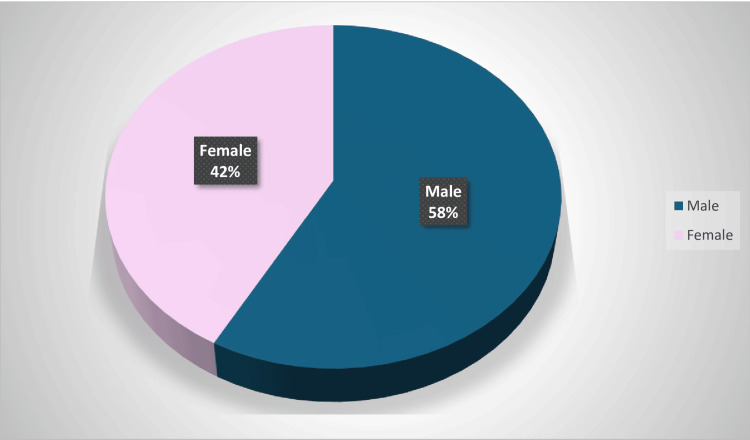
Sex distribution.

**Figure 6:**
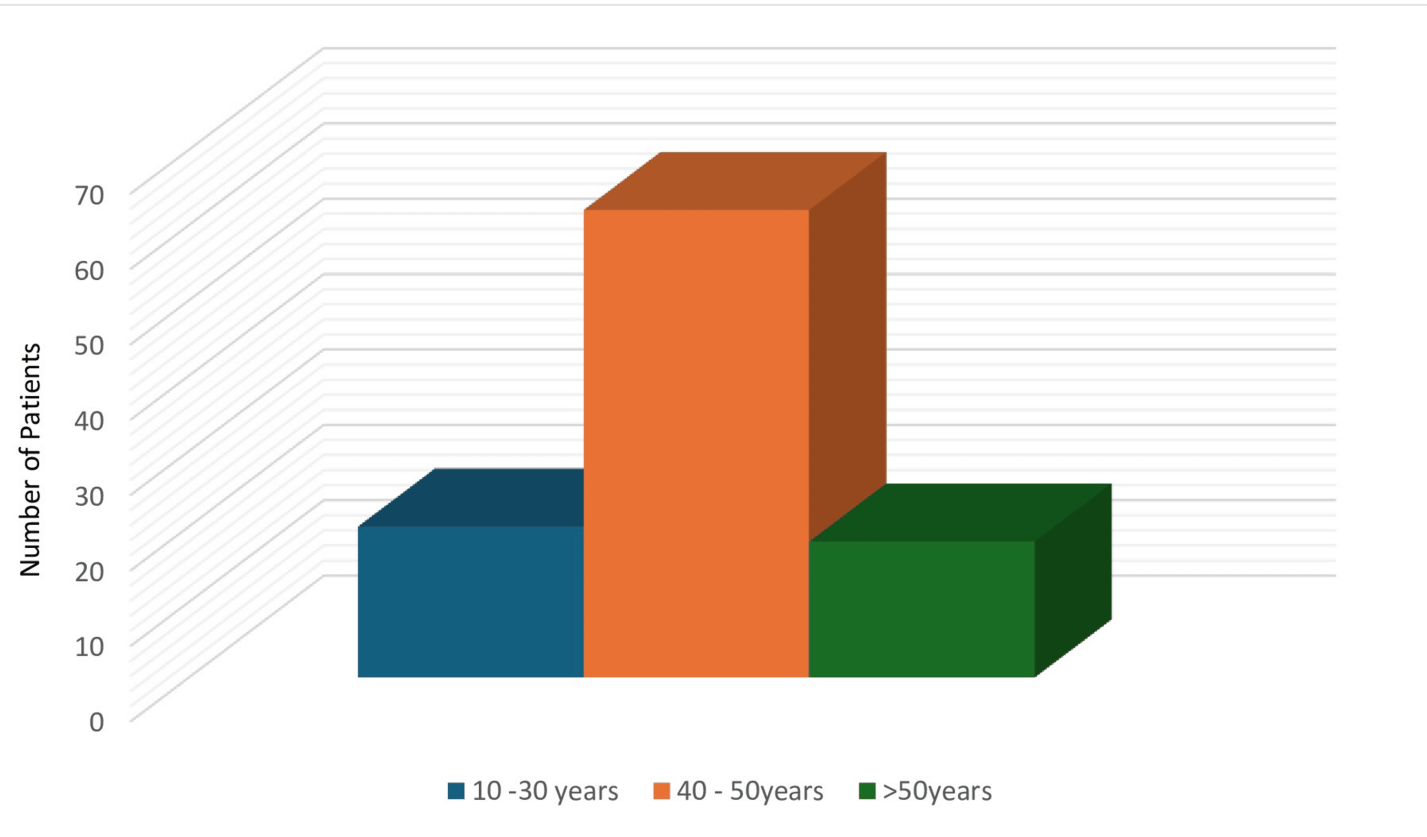
Age distribution. Most patients were aged 40-50 years. Data are represented by frequency per age group.

**Figure 7:**
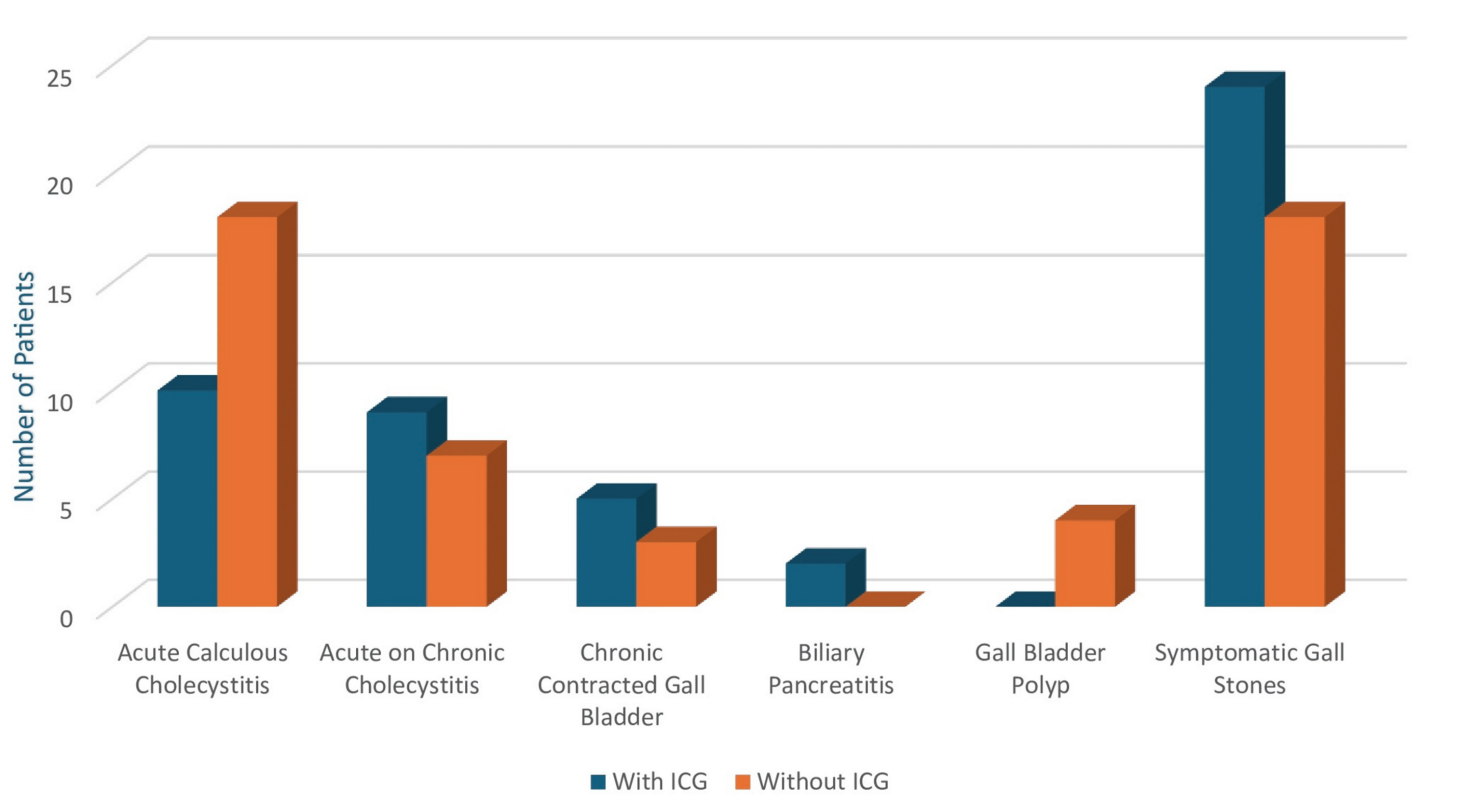
Case distribution. Distribution of gallbladder diagnoses among ICG-assisted and non-ICG groups (N = 100). P-values were calculated using the chi-square test. All comparisons were not statistically significant (p > 0.05). NS: Not significant; ICG: Indocyanine green.

Among the 100 cases analyzed, 42 were female and 58 were male (Figure 5). The majority of patients belonged to the 40-50-year age group (Figure 6). Regarding case distribution, 28 (28%) were diagnosed with acute calculous cholecystitis, of which 10 (10%) underwent surgery with ICG and 18 (18%) without. Acute-on-chronic cholecystitis was observed in 16 (16%) of cases (9 (9%) with ICG and 7 (7%) without), while 8 (8%) had a chronically contracted gallbladder (5 (5%) with ICG and 3 (3%) without). Additionally, 42 (42%) presented with symptomatic gallstone disease (24 (24%) with ICG and 18 (18%) without), while the remaining cases included gallbladder polyps and biliary pancreatitis, which were not treated using ICG (Figure 7).

The duration of surgery, measured from the start of dissection to port closure, was slightly shorter in cases where ICG was used compared to those without ICG. The mean operative time was 42.6 ± 5.2 minutes in the ICG group versus 48.3 ± 6.1 minutes in the non-ICG group. The observed difference in mean operative times was associated with a p-value of 0.05, which is at the conventional threshold for statistical significance. Therefore, while the reduction is modest, it may be considered marginally significant (Table 1). 

**Table 1:** Duration of surgery. Comparison of mean operative time between ICG-assisted laparoscopic cholecystectomy (n = 50) and standard laparoscopic cholecystectomy (n = 50). Data are presented as mean ± SD. P-values were derived from an independent t-test. A p-value < 0.05 was considered statistically significant. ICG: Indocyanine green.

Group	N	Minimum	Maximum	Mean	SD	P-value
With ICG (Duration of Surgery)	50	32	50	42.6	5.2	0.05
Without ICG (Duration of Surgery)	50	36	60	48.3	6.1	0.05

Intraoperatively, no bile duct or bowel injuries were encountered, and all procedures were successfully completed without the need for conversion to open surgery or bailout interventions.

Excessive bleeding (>100 mL) during dissection was noted in 6 (6%) cases, 4 (4%) in the ICG group and 2 (2%) in the non-ICG group. These cases predominantly involved patients with a severely inflamed gallbladder, which may have contributed to a prolonged operative time. However, the difference in bleeding incidence between the two groups was not statistically significant (p = 0.48; 95% CI: -0.19 to 0.07).

Regarding hospital stay, the median duration was 1 day (range: 1-2 days) for patients in the ICG group and 1.3 days (range: 1-2 days) for those without ICG. The reduction in hospital stay was statistically significant (p = 0.045; 95% CI: -0.58 to -0.01 days). No cases of bile leak were reported during the postoperative period (Table 2).

**Table 2:** Descriptive statistics for duration of surgery. Descriptive statistics comparing the duration of surgery between ICG-assisted and non-ICG groups. P-values were calculated using an independent t-test; a p-value < 0.05 was considered statistically significant. ICG: Indocyanine green.

Statistic	Without ICG	With ICG
Mean (minutes)	39.28	38.34
Standard Error	0.753	0.758
95% CI Lower Bound	37.77	36.82
95% CI Upper Bound	40.79	39.86
5% Trimmed Mean	39.34	38.62
Median (minutes)	40	39
Variance	28.369	28.76
SD	5.326	5.363
Minimum (minutes)	25	25
Maximum (minutes)	50	47
Range	25	22
IQR	7	8
Skewness	-0.268	-0.714
Skewness Std. Error	0.337	0.337
Kurtosis	-0.009	0.372
Kurtosis Std. Error	0.662	0.662

## Discussion

LC is the gold standard for gallbladder removal, offering a minimally invasive approach with reduced postoperative pain, shorter hospital stays, and faster recovery [2]. However, despite its advantages, BDI remains a serious concern, with an incidence of 0.3%-0.7%, even among experienced surgeons [7,12]. These injuries can lead to severe complications, including biliary strictures, cholangitis, liver dysfunction, and the need for complex reconstructive surgery [6,15].

To mitigate this risk, intraoperative biliary visualization techniques such as IOC, laparoscopic ultrasound, and near-infrared fluorescence cholangiography (NIR-FC) with ICG have been developed [8,13]. Among these, ICG fluorescence cholangiography has gained increasing attention as a radiation-free, real-time, and dynamic imaging modality that enhances visualization of biliary anatomy without requiring contrast injection into the bile ducts [10,14]. In our study, we aimed to assess whether ICG-assisted LC provides a tangible advantage over conventional LC, focusing on key parameters such as operative time, BDI rate, conversion rate, and overall safety profile.

Our findings suggest that while ICG fluorescence imaging did not significantly reduce the incidence of bile duct injuries-since none occurred in either group-it notably shortened operative duration in the ICG group compared to non-ICG cases [10,11]. This is likely due to the ability of ICG to provide early and precise delineation of the cystic duct, common bile duct (CBD), and cystic artery, thereby enabling more efficient and safer dissection within Calot’s triangle [13,14]. Similar observations have been reported in studies demonstrating that ICG fluorescence significantly improves biliary mapping, particularly in cases of acute inflammation or anatomical variations [16,17].

Comparing our findings with previously published research, studies have shown that ICG fluorescence cholangiography improves biliary visualization and reduces operative time while maintaining a low BDI rate [4,13,17]. This aligns with our results, reinforcing its role as a valuable adjunct in LC. However, potential confounding factors such as surgeon experience and patient comorbidities should be considered. Our study was conducted by a single surgeon with ten years of experience in LC, which could have influenced the safety outcomes. Additionally, patient comorbidities-including obesity, diabetes, and prior abdominal surgeries-may impact the effectiveness of ICG imaging and overall surgical outcomes [1,2,17].

BDI prevention remains a key objective in LC, as its consequences can lead to long-term morbidity and complex reoperative surgery [7,9]. Studies suggest that ICG fluorescence cholangiography enhances the identification of biliary structures, particularly in difficult dissections where traditional white-light laparoscopy may be insufficient. Additionally, NIR-FC has been shown to be superior to standard visualization techniques in cases with distorted biliary anatomy, such as acute cholecystitis or previous upper abdominal surgeries [3,16]. This aligns with our findings, where ICG fluorescence significantly enhanced visualization, leading to quicker and more confident identification of biliary structures.

Furthermore, ICG fluorescence cholangiography provides a dynamic, real-time view of the extrahepatic biliary tree, distinguishing bile ducts from surrounding tissues without requiring mechanical manipulation of the cystic duct for contrast injection, as is the case with IOC [17]. This is particularly advantageous in cases where Calot’s triangle is obscured by inflammation or adhesions-conditions that increase the risk of BDI during dissection.

Despite these advantages, feasibility and cost-effectiveness must be considered. Our findings indicate that ICG fluorescence cholangiography does not impose a significant financial burden on institutions or patients. The dye is relatively inexpensive, and the required near-infrared imaging system can be integrated into existing laparoscopic setups without excessive cost [5,11,15]. However, barriers to widespread adoption include the availability of technology and the expertise required to assess and utilize it effectively. Training programs and institutional investments in near-infrared imaging systems will be necessary to facilitate broader implementation of this technique.

Another key consideration is the optimal timing and dosage of ICG administration. Studies indicate that variations in ICG dose and timing can impact fluorescence intensity and visualization quality, influencing the surgeon's ability to delineate biliary anatomy effectively [11,15]. While intravenous ICG injection one hour before surgery is the most commonly used protocol, recent research has explored direct gallbladder injection of ICG as an alternative approach with promising results [11]. Standardizing these protocols could further enhance the effectiveness of ICG fluorescence cholangiography in routine surgical practice.

Despite these challenges, our findings, along with prior studies, suggest that ICG fluorescence imaging should be considered a valuable adjunct to LC. While it may not replace IOC in complex cases, it provides a non-invasive, radiation-free, and efficient method for enhancing biliary visualization, thereby potentially reducing operative time and improving surgical safety [16].

Moving forward, larger multicenter trials involving diverse surgical teams will be essential to determine the optimal clinical applications of ICG fluorescence imaging and its role in establishing standardized guidelines for biliary visualization in LC [16].

This study has several limitations. First, it was a single-center study conducted by a single surgeon, which may limit the generalizability of the findings. The operator's skill level and familiarity with ICG technology likely influenced the results, particularly regarding operative time and complication rates. Second, the sample size was relatively small (N = 100), which may reduce the power to detect rare events such as bile duct injuries. Third, long-term follow-up was not conducted to assess delayed complications, such as biliary strictures or reinterventions. Additionally, variables such as BMI, previous abdominal surgeries, and comorbid conditions were not stratified or adjusted for, which could affect surgical difficulty and outcomes. Lastly, cost-effectiveness analysis was descriptive and not formally evaluated using economic modeling. Although this study did not include a formal cost-effectiveness analysis, the use of ICG is considered affordable, and the imaging equipment is increasingly integrated into modern laparoscopic systems. Future multicenter trials should incorporate detailed economic modeling to assess cost-effectiveness in terms of reduced operative time, complications, and hospital stay. Addressing these limitations in future studies will help provide a more comprehensive evaluation of ICG fluorescence cholangiography.

## Conclusions

LC with real-time ICG fluorescence cholangiography enhances intraoperative biliary visualization, contributing to safer and more efficient surgeries. This study supports the integration of ICG as a valuable adjunct to conventional techniques, offering potential benefits in operative workflow and postoperative recovery. The absence of complications further reinforces its favorable safety profile. Broader adoption of ICG imaging may be supported by standardizing its application and evaluating its impact across diverse clinical settings and levels of surgical expertise.
